# Short-term moderate diet restriction in adulthood can reverse oxidative, cardiovascular and metabolic alterations induced by postnatal overfeeding in mice

**DOI:** 10.1038/srep30817

**Published:** 2016-07-28

**Authors:** Na Li, Charles Guenancia, Eve Rigal, Olivier Hachet, Pauline Chollet, Lucie Desmoulins, Corinne Leloup, Luc Rochette, Catherine Vergely

**Affiliations:** 1 Inserm UMR866, Laboratoire de Physiopathologie et Pharmacologie Cardio-Métaboliques (LPPCM), Université de Bourgogne Franche-Comté, Dijon, France; 2 Service de Cardiologie, Centre Hospitalier Universitaire, Dijon, France; 3 CNRS UMR 6265, Centre des Sciences du Goût de l’Alimentation, Dijon, France; 4 INRA UMR 1324, Centre des Sciences du Goût de l’Alimentation, Dijon, France; 5 Univ. Bourgogne Franche-Comté, UMR Centre des Sciences du Goût de l’Alimentation Dijon, France

## Abstract

We aimed to determine whether moderate diet restriction could restore cardiac, oxidative and metabolic alterations induced by postnatal overfeeding (PNOF). Litters of C57BL/6 male mice were either maintained at 9 (normal litter, NL), or reduced to 3 (small litter, SL) in order to induce PNOF. At 6 months, half of the NL and SL mice were subjected to 20% calorie-restriction (CR: NLCR, SLCR) for one month, while the other half continued to eat ad libitum (AL: NLAL, SLAL). Six-month old SL mice presented overweight, fat accumulation, hyperleptinemia, glucose intolerance, insulin resistance, increased cardiac ROS production and decreased left ventricular ejection fraction (LVEF). After CR, SL mice body weight was normalized; however, their fat mass and leptinemia were not decreased, glucose metabolism was improved and LVEF was increased. In SL mice, CR increased the cardiac mitochondrial respiratory rate and decreased cardiac ROS production. Hearts from SLCR mice showed better recovery and smaller postischemic infarct size. Intriguingly, no difference was observed between NLAL and NLCR mice for most of the parameters investigated. Short-term moderate CR not only normalized body weight in SL mice but also improved metabolic programming and reversed oxidative and cardiac dysfunction induced by PNOF.

Clinical and experimental evidence indicates that the environment during the perinatal period and early development plays a key role in regulating metabolic tendencies in adulthood. Epidemiological studies have shown that early nutritional conditions may permanently affect future body weight and cardio-metabolic risk factors such as hypertension, dyslipidemia, and insulin resistance, which ultimately affect the propensity to develop cardiovascular diseases^1^,^2^,^3^,^4^,^5^. Indeed, due to genomic plasticity, the neonatal period is critical for the orientation of phenotypic features in adulthood, since epigenetic modifications may impact gene expression and influence the development of chronic disease.

Early overnutrition can be induced in mice and rats by reducing litter size^6^,^7^, a situation that reduces competition for milk during the suckling period and therefore leads to over-nourishment in the postnatal period^8^. Several studies have shown that litter size reduction in rodents leads to substantial changes in body weight at weaning (nearly 30% increase), which persisted at a lower level in mature animals^9^,^10^,^11^. A cluster of symptoms characteristic of the metabolic syndrome, like overweight, raised blood pressure and insulin resistance, have been observed in postnatally overfed rats^12^,^13^. In addition, concerning cardiac development, overnutrition in early life induces cardiac hypertrophy and reduces myocardial vessel density in young rodents^14^. Impaired insulin signaling in the heart of adult postnatally overfed rat has also been reported^15^,^16^. Previous research performed in our team has shown that overnutrition during the immediate postnatal period in rats and mice leads to early changes in cardiac gene expression, which may permanently modify the heart’s structural organization and metabolism and could impair cardiac contraction and increase susceptibility to myocardial ischemia-reperfusion injury^10^,^17^,^18^ and to anticancer drugs cardiotoxicity^19^.

Calorie restriction (CR) is defined as a reduction in calorie intake below the usual ad libitum (AL) intake without malnutrition. Evidence from animal studies and from a limited number of human trials indicates that CR has the potential to both delay cardiac aging and help prevent atherosclerotic cardiovascular disease via its beneficial effects especially on blood pressure, lipids, inflammatory and redox processes^20^,^21^. The magnitude of CR applied in most rodent studies varies between severe with a 40–50% CR, and moderate with a 20–25% CR^22^. In a rat model of overweight induced by high-carbohydrate milk formula, appropriate manipulation of energy intake during the early post-weaning period restored body weight and normalized serum insulin and leptin concentrations^23^. A moderate CR 24% after weaning could also lead to longer-lasting effects on the regulation of body weight by reversing the effects of early pre-weaning programming on the hypothalamic regulation mechanism^24^. However, to date, no study has extensively explored, in mature rodents, the effects of body weight normalization on the cardio-metabolic risk induced by postnatal programming.

Additionally, while mitochondria provide an essential source of energy to drive cellular processes and are particularly important in heart muscle cells, they are also a major source of reactive oxygen species as a by-product of the respiratory chain. Interestingly, in a model of perinatal overnutrition induced by a maternal obesogenic diet, myocardial and mitochondrial ultrastructural abnormalities were observed in offspring, suggesting that this organelle may play an essential role in translating the stress associated with early nutritional programming into cardiac dysfunction in later-life^25^.

The aim of our study was therefore to determine whether normalization of body weight by moderate CR in six-month-old mice might reverse cardiac and metabolic alterations induced by postnatal overfeeding (PNOF), and to investigate the potential involvement of cardiac mitochondrial oxidative stress in this context.

## Results

### Body Weight and Body Composition

**Figure 1:**
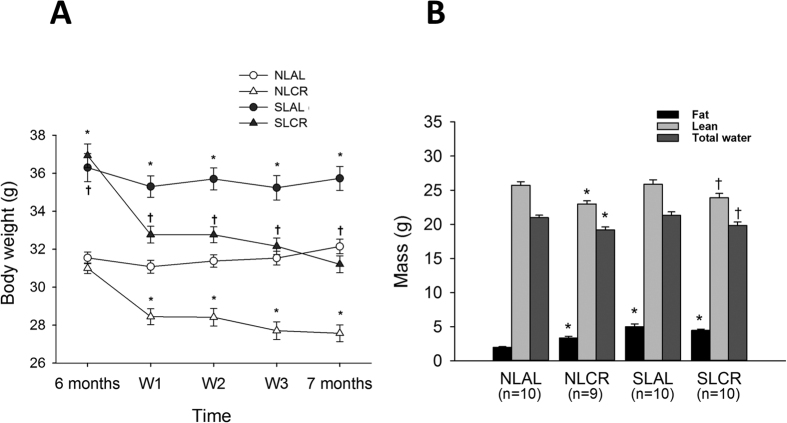
Evolution of body weight (A) and body composition (B) of control mice raised in normal litters (NL) or postnatally overfed mice raised in small litters (SL) which, at 6 months of age, were assigned to be fed either ad libitum (AL) or with 20% calorie restriction (CR) for 1 month, until they attained 7 months of age. Four groups were then constituted: NLAL (n = 30), NLCR (n = 30), SLAL (n = 29) and SLCR (n = 33). *significantly different from NLAL, p < 0.001. ^†^significantly different from SLAL, p < 0.001. Body composition was measured by nuclear magnetic resonance, and expressed as mass of fat, lean and free water from body weight in 7-month-old NLAL (n = 10), SLAL (n = 10), NLCR (n = 9) and SLCR (n = 10) mice.

During the neonatal suckling period, weight gain was significantly greater in pups raised in small litters (SL). At weaning (day 24), the body weight of SL mice was 30% greater than that of normal litter (NL) mice (NL (n = 60): 9.7 ± 0.9 g; SL (n = 62): 12.9 ± 1.3 g, p < 0.001). This difference persisted during growing and maturity, but to a lesser extent: at 6 months of age, the body weight of SL mice was 17% greater than that of the NL group (Fig. 1A, 36.63 ± 0.58 g vs. 31.33 ± 0.28 g, p < 0.001). Analysis of food intake revealed that, at 6 months, SL mice consumed nearly 15–20% more chow per day on average as compared with NL mice (Table 1). Four weeks of moderate (20%) CR significantly reduced the body weight of SL mice to a level comparable to that of NL mice fed ad libitum (AL) (Fig. 1A). A similar level of CR in control NL mice (NLCR) also decreased their body weight significantly. The higher body weight in SL mice was associated with a significantly greater percentage of fat mass (Fig. 1B, 14.5% ±1.2 in SLAL vs. 6.3% ±0.4 NLAL, p < 0.001). Intriguingly, although 4 weeks of CR decreased global body weight, it did not modify the percentage of fat mass in SLCR mice (13.8% ±0.7), and surprisingly it increased the fat mass percentage in lean NLCR mice (11.0% ±0.9). The lean mass was decreased in NLCR mice as compared to NLAL (25.71 g ±1.58, vs. 22.96 g ±1.44, p < 0.01) and in SLCR as compared to SLAL (25.86 g ±2.19, vs. 23.89 g ±2.00, p < 0.01).

### Glucose and Insulin tolerance test (GTT and ITT)

**Figure 2:**
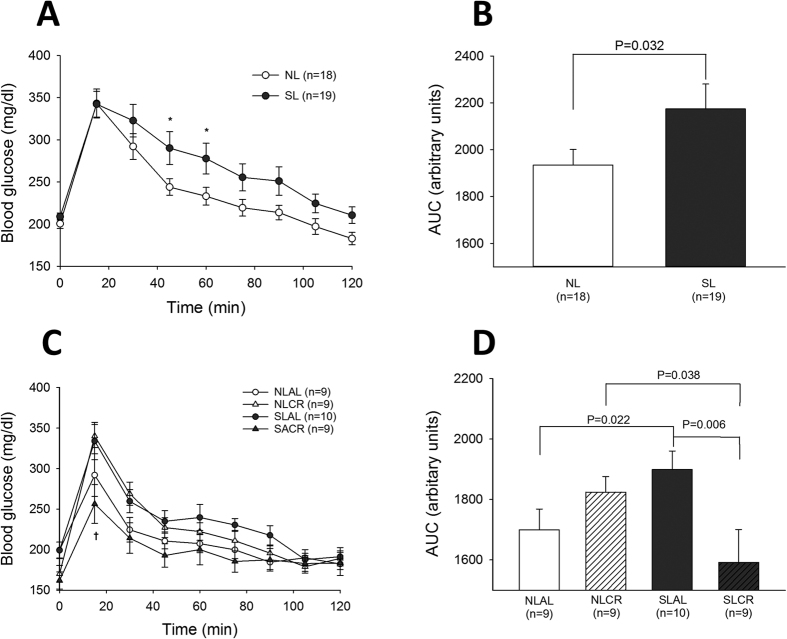
Intraperitoneal Glucose Tolerance Test (ipGTT) in control mice raised in normal litters (NL, n = 18) or postnatally overfed mice raised in small litters (SL, n = 19) which, at 6 months of age, were assigned to be fed either ad libitum (AL) or with 20% calorie restriction (CR) for 1 month, until they attained 7 months of age (4 groups were constituted: NLAL (n = 9), NLCR (n = 9), SLAL (n = 10) and SLCR (n = 9)). Evolution blood glucose (**A,C**) and area under the curve of blood glucose (**B,D**) after glucose (2 g/kg) administration at 6 months of age before CR (**A,B**), and after 4 weeks of CR (**C,D**) *significantly different from NL/NLAL, p < 0.05. ^†^significantly different from SLAL, p < 0.001.

**Figure 3:**
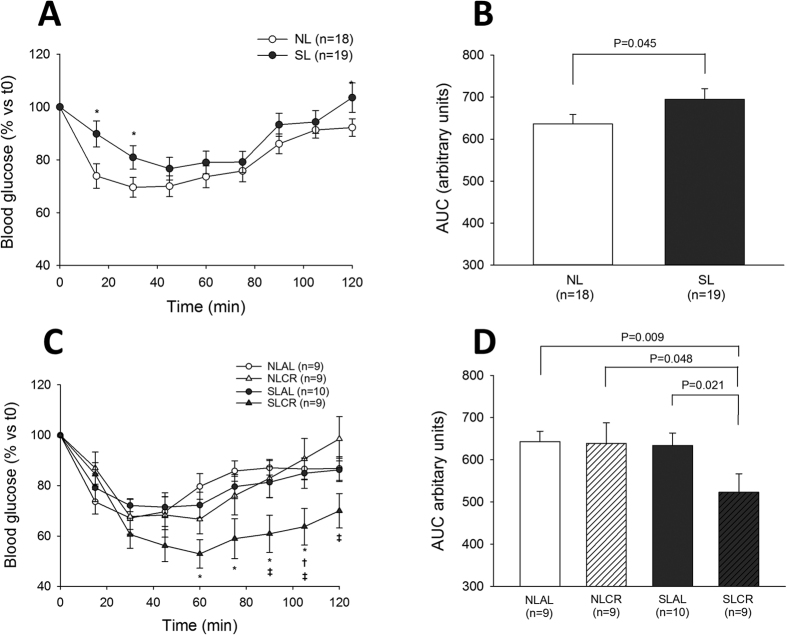
Intraperitoneal Insulin Tolerance Test (ipITT) in control mice raised in normal litters (NL, n = 18) or postnatally overfed mice raised in small litters (SL, n = 19) which, at 6 months of age, were assigned to be fed either ad libitum (AL) or with 20% calorie restriction (CR) for 1 month, until they attained 7 months of age (4 groups were constituted: NLAL (n = 9), NLCR (n = 9), SLAL (n = 10) and SLCR (n = 9)). Evolution of blood glucose (**A,C**) and area under the curve for blood glucose (**B,D**) after insulin (0.75 IU/kg) administration at 6 months of age before CR (**A,B**), and after 4 weeks of CR (**C,D**) *significantly different from NL/NLAL, p < 0.05. ^†^significantly different from SLAL, p < 0.001. ^‡^significantly different from NLCR, p < 0.001.

In order to assess glucose tolerance and insulin resistance in the different groups of mice, intraperitoneal ITT and GTT were performed before and after CR. At 6 months of age, blood glucose concentrations were significantly higher in SL than in NL mice 45 min and 60 min after glucose injection (Fig. 2A), and the area under curve (AUC) of blood glucose concentration was also greater in the SL group (Fig. 2B). SL mice that continued to eat AL for 4 weeks remained glucose-intolerant (Fig. 2D); however, CR significantly decreased blood glucose levels in SL mice but not in NL mice (Fig. 2D). ITT performed at 6 months revealed that the decrease in blood glucose after insulin injection was significantly impaired in SL mice (Fig. 3A,B), showing insulin resistance in this group. Sensitivity to insulin was not only restored but also amplified by CR in SL mice (Fig. 3C,D), whereas CR did not affect insulin sensitivity in NL mice.

### Plasma levels of glucose, insulin and leptin

Fasting plasma glucose (Table 2) levels were similar in NL and SL mice, but significantly lower in both NLCR and SLCR mice than in their respective AL group. Fasting insulin levels were significantly higher in SLAL mice, whereas SLCR mice had levels similar to those in NL groups. Calculated HOMA-IR followed the evolution of insulin levels in the four groups. Fasting leptin levels were significantly higher in SLAL than in NLAL mice. Interestingly, leptin levels were not modified by CR in SL mice, and were even increased in NL mice. Plasma adiponectin levels were not different among the four groups.

### *In vivo* Cardiac Function

**Figure 4:**
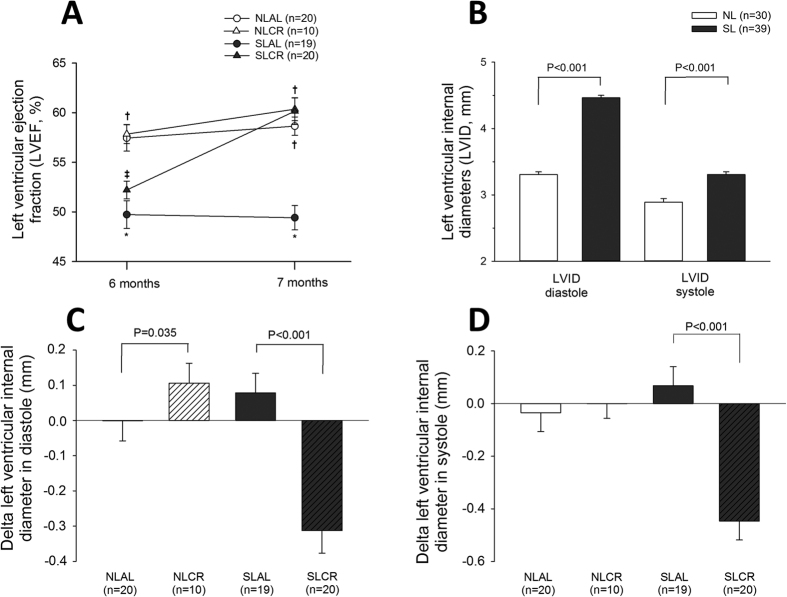
Cardiovascular function assessed by echocardiography in anesthetized mice. Control mice were raised in normal litters (NL, n = 30) and postnatally overfed mice raised in small litters (SL, n = 39) and, at 6 months of age, when mice were assigned to be fed either ad libitum (AL) or with 20% calorie restriction (CR) for 1 month, until they attained 7 months of age (4 groups were constituted: NLAL (n = 20), NLCR (n = 10), SLAL (n = 19) and SLCR (n = 20)). (**A**) Evolution of left ventricular ejection fraction (LVEF, in %). (**B**) Left ventricular internal diameters (LVID) in diastole and in systole at 6 months. (**C,D**) Changes in left ventricular internal diameters in diastole (**C**) and systole (**D**) between 6 and 7 months. *significantly different from NLAL, p < 0.05. ^†^significantly different from SLAL, p < 0.05. ^‡^significantly different from NLCR, p < 0.05.

The echocardiographic parameters measured in mice at 6 and 7 months old are presented in Table 3. The left ventricular ejection fraction (LVEF) was significantly lower in 6-month-old SL mice than in NL mice; however there were no changes in the diastolic function or modification of LV mass in SL mice. After AL for 4 weeks, LVEF remained unchanged in NL and SL mice (Fig. 4A). CR did not modify the left ventricular (LV) systolic function in the NL group. However, in SL mice, 4 weeks of CR led to a significant increase in LVEF (52% ±4 vs. 60% ±6, p < 0.001). Additionally, left ventricular internal diameters (LVID) in diastole and systole, initially greater in SL mice than in NL mice at 6 months old (Fig. 4B), were significantly decreased in SL mice after 4 weeks of CR (Fig. 4C). The heart to body weight ratio, a measurement of heart hypertrophy, was similar in all groups.

### Heart’s Susceptibility to Ischemia/Reperfusion (I/R) Injury

**Figure 5:**
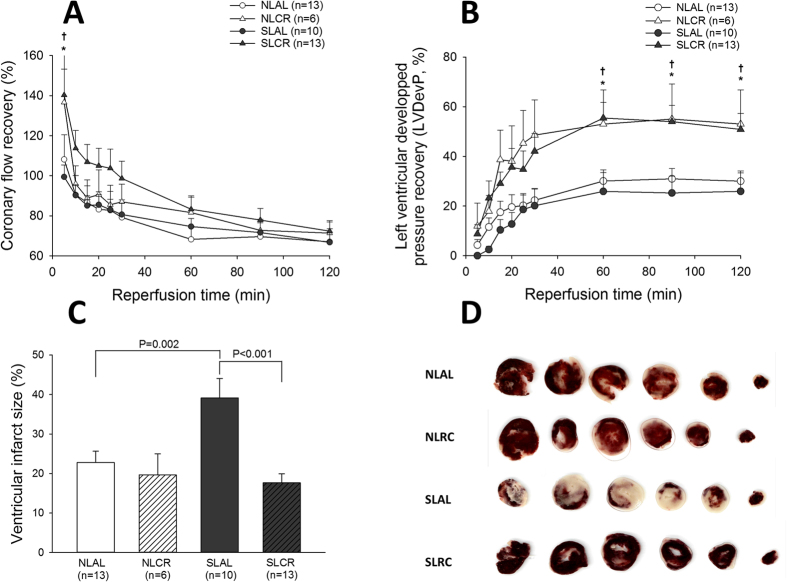
Myocardial recovery during reperfusion after 30 min of global ischemia of hearts isolated from 7-month-old control mice raised in normal litters (NL) and postnatally overfed mice raised in small litters (SL) which, at 6 months of age, were assigned to be fed either ad libitum (AL) or with 20% calorie restriction (CR) for 1 month (4 groups were constituted: NLAL (n = 13), NLCR (n = 6), SLAL (n = 10) and SLCR (n = 13)). (**A**) Recovery of pre-ischemic coronary flow (in %). (**B**) Recovery of pre-ischemic developed pressure (in %) (**C**) Ventricular infarct size (% of ventricular surface). (**D**) Representative TTC staining of heart slices. *significantly different from NLAL, p < 0.05. ^†^significantly different from SLAL, p < 0.05.

In isolated hearts of 7-month-old mice, during baseline conditions of perfusion, there was no difference between the four groups for all of the cardiac parameters measured (data not shown). After 30 min of global myocardial ischemia, reperfusion led to progressive but incomplete recovery of the heart’s circulating and contractile activity (Fig. 5A). There was no difference between NLAL mice and SLAL mice for the recovery of coronary flow and of left ventricular developed pressure (LVDP). However, the recovery of LVDP was significantly better in the NLCR and SLCR mice at any time point during reperfusion in comparison with their AL control group (Fig. 5B). The evaluation of viable (red) and necrotic zones (white) after 2 h of reperfusion (Fig. 5D) showed a greater infarcted area in SLAL hearts than in hearts from any other group. In particular, it was significantly greater than that in SLCR mice (Fig. 5C).

### Oxidative Stress in cardiac tissues

**Figure 6:**
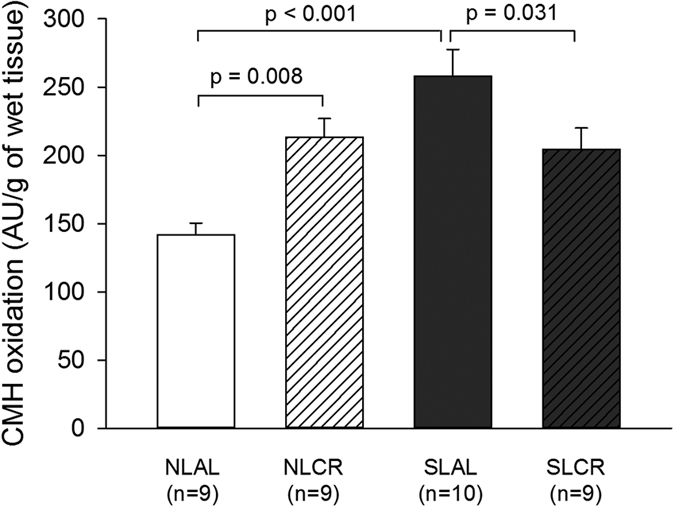
Formation of reactive oxygen/nitrogen species (RONS) in heart tissue from 7-month-old control mice raised in normal litters (NL) and postnatally overfed mice raised in small litters (SL) which, at 6 months of age, were assigned to be fed either ad libitum (AL) or with 20% calorie restriction (CR) for 1 month (4 groups were constituted: NLAL (n = 9), NLCR (n = 9), SLAL (n = 10) and SLCR (n = 9)). Formation of ROS was evaluated with electron spin resonance spectroscopy by the oxidation of the cell-permeable CMH hydroxylamine probe into CP^●^ nitroxide radical.

Cardiac oxidative stress levels were measured in freshly harvested left ventricular tissue (at the apex) by electron spin resonance (ESR) spectroscopy (Fig. 6). A characteristic anisotropic signal corresponding to the oxidation of 1-hydroxy-3-methoxycarbonyl-2,2,5,5- tetramethyl pyrrolidine (CMH) into CP^•^ nitroxide radical by reactive oxygen-nitrogen species (RONS) was observed. After quantification of the signals, we found a far higher level of CMH oxidation in myocardial biopsies from SL mice. Puzzlingly, CR had opposite effects in NL and SL mice, with a decrease in CMH oxidation in SLCR mice and an increase in NLCR mice. However, antioxidant enzyme gene expression in the heart ventricular tissue from 7-month-old mice was identical in all mice (Table 4).

### Mitochondrial Function in cardiac fibers

**Figure 7:**
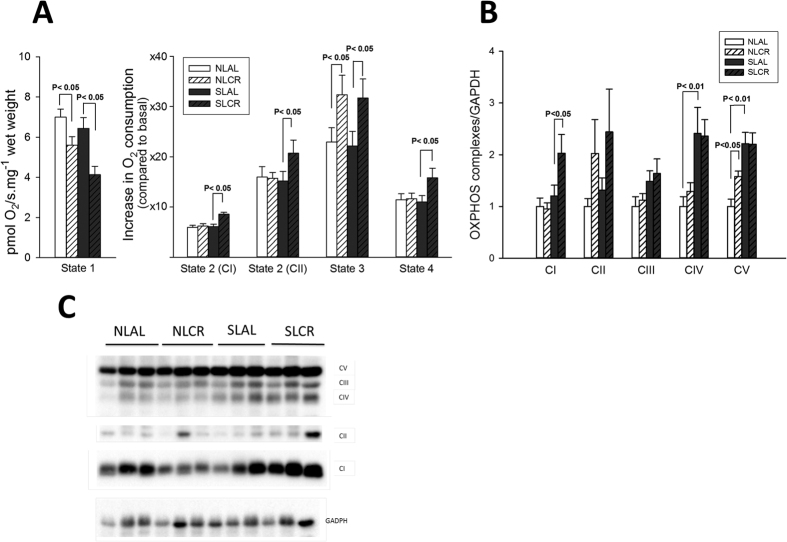
Mitochondrial respiratory chain function from 7-month-old control mice raised in normal litters (NL) and postnatally overfed mice raised in small litters (SL) which, at 6 months of age, were assigned to be fed either ad libitum (AL) or with 20% calorie restriction (CR) for 1 month (4 groups were constituted: NLAL, NLCR, SLAL and SLCR). (**A**) Respiratory flux rates of permeabilized cardiac fibers. The respiratory parameters are: State 1, Basal oxygen consumption of mitochondria with endogenous substrates; State 2:, glutamate + malate + pyruvate (substrates-driven respiration through CI); State 2: by adding succinate (substrate-driven respiration through CII); State 3: further adding ADP (maximal substrates-ADP driven respiration: maximal coupled respiration); State 4: inhibition of CV through carboxy-atractyloside, allowing the measurement of non-coupled respiration of coupled mitochondria (maximal leak). (**B**) Cardiac expression of the five complexes (CI-CV) of the respiratory chain (OXPHOS) as determined by immunoblotting. (**C**) Representative immunoblotting of protein levels of OXPHOS complexes I-V. Cardiac protein level was normalized to glyceradehyde-3-phosphate dehydrogenase (GAPDH) content and expressed relative to the control (NL) group.

Given that contractile function is influenced by oxidative energy metabolism, oxygen consumption by mitochondria was measured using a respirometer on permeabilized papillary muscle. Concerning the basal oxygen consumption (state 1), there was no difference between NLAL and SLAL groups. However, basal oxygen consumption (state 1) in NLCR and SLCR was lower than that in their respective AL groups (Fig. 7A). For state 2 (substrate-driven respiration), we performed titrations with glutamate, malate and pyruvate in order to stimulate the respiration with complex I (CI, NADH dehydrogenase) succinate in order to assess the respiration with complex II (CII, succinate dehydrogenase). State 3 respiration (maximal coupling respiration) was measured with saturating ADP concentration in order to assess maximal oxidative phosphorylation (OXPHOS). CAtr, an ATP-ADP exchange inhibitor, was then added to block complex V and obtain the ADP-independent resting state 4, when respiration is only driven by substrates. There was no difference between NLAL and SLAL mice for any state of mitochondrial respiration, but we systematically observed a greater increase in oxygen flux consumption in SLCR mice than in SLAL mice after titrations with all substrates. Additionally, O_2_ flux consumption was higher in both NLCR mice and SLCR mice than in their AL controls. However, the respiration control ratio (state3/state 4), an index of OXPHOS efficiency, was similar in the four groups, due to an almost equal alteration of each state.

Expression of the five mitochondrial complexes was then examined by Western blotting (Fig. 7B,C). The expression of complexes IV and V was greater in hearts from SLAL mice than in hearts from NLAL mice (Fig. 7B). CR induced enhanced expression of complex I in SL mice, and of complex V in NL mice.

### Signal transducer and activator of transcription 3 (STAT3) Signaling in the heart

**Figure 8:**
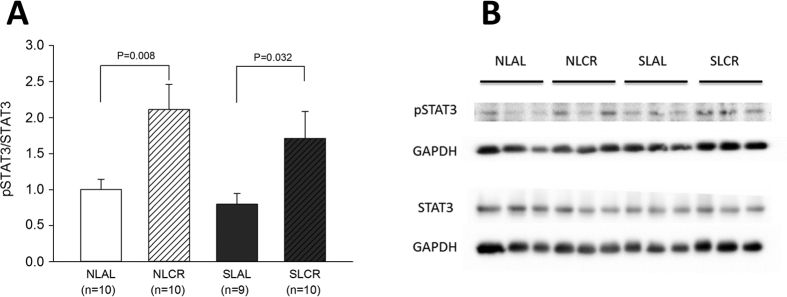
Cardiac phosphorylation level of STAT3 evaluated in 7-month-old control mice raised in normal litters (NL) and postnatally overfed mice raised in small litters (SL) which, at 6 months of age, were assigned to be fed either ad libitum (AL) or with 20% calorie restriction (CR) for 1 month (4 groups were constituted: NLAL (n = 10), NLCR (n = 10), SLAL (n = 9) and SLCR (n = 10)). (**A**) Cardiac expression of pSTAT3/STAT3 ratio as determined by immunoblotting of phosphorylated form and total form of the protein. The ratio of their respective levels is indicative of the activation. (**B**) Representative immunoblotting of protein levels.

The JAK/STAT pathway is at the crossroads of several signaling pathways, involving growth factors receptors, receptors of cytokines such as IL-6, G-protein coupled receptors and TLR; however, it was interesting to evaluate its activation in the hearts of our mice since it is involved both in leptin signaling and in cardioprotection. The activation of leptin receptor (Ob-Rb) by its ligand leads to JAK activation followed by tyrosine phosphorylation of STAT3 protein, which then translocates to the nucleus in order to activate transcription of target genes. Additionally, one of the cardioprotective pathways involved in ischemic preconditioning, the Survivor Activating Factor Enhancement (SAFE) pathway, requires the activation of STAT3^26^. Interestingly, the pSTAT3/STAT3 ratio in hearts from NLCR and SLCR mice was greater than that in their respective AL groups (Fig. 8).

## Discussion

Clinical and experimental data from animal models have clearly demonstrated that altered nutritional experiences during critical periods of development are an important factor in the etiology of obesity and related metabolic diseases. Previous research at our laboratory indicated that postnatal overnutrition induced not only metabolic symptoms, but also early changes in heart gene expression that may have a long-term effect on cardiovascular function and heart structure in adult rodents, and may have a deleterious impact on myocardial recovery after ischemic injury^10^. The deterioration in cardiovascular function in adulthood could be either a direct effect of early programming by postnatal overnutrition, or explained by the side-effects of adult-onset overweight and modifications in the metabolic environment in adulthood, or both. A healthy lifestyle is one of the most effective therapies for metabolic syndrome. In fact, increased physical activity combined with nutritional advice is the first line of treatment as recommended by international guidelines^27^.

The major aim of the study was to determine whether this programmed increase in cardio-metabolic risk induced by perinatal programming, could be, in a certain way, “de-programmed” by a short-term nutritional adjustment, at a time when the phenotype seems to be definitely acquired. We hypothesized that normalization of body weight by moderate CR could reverse or improve cardio-metabolic complications induced by postnatal over-nutrition. For this purpose, 4 weeks of moderate (−20%) CR was imposed on 6-month-old male SL mice, in order to decrease the body weight of SL mice to that of NL mice.

In the present study, we found that postnatal over-nutrition induced a significant increase in body weight at weaning, which persisted at a lower level with maturation. This finding is consistent with previous studies. This increase in body weight was explained by an increase in body fat mass, at the expense of lean mass^10^. An increase in visceral white adipose tissue is observed at weaning and later in SL rats^8^,^28^ and mice^29^ and is accompanied by an increase in adipocyte surface^6^,^13^,^30^. However, subcutaneous fat mass is also increased in young but not adult SL mice^31^, indicating that in adulthood, higher body weight and fat mass are mainly due to increased visceral fat mass. Interestingly, our moderate CR diminished body weight in both SL and NL mice; however, it did not have the same effect on fat metabolism in the two groups. After 4 weeks of CR, the fat mass percentage in SL mice remained stable while that of NL mice increased significantly. This finding is novel, in that it suggests that during moderate CR in mice, the adipose tissue is preserved, and that weight loss is rather due to a reduction in lean mass. The leptin levels corroborate this finding: SLCR mice showed no change in leptin levels, suggesting that adipocyte endocrine function, which is mainly related to fat mass, was also unchanged. Studies performed by other authors, using much more severe and longer CR (commonly 40% for 3 months) showed decreased fat mass^32^. This suggests that the moderate CR used in our study may be too short or insufficiently harsh to affect fat metabolism. Additionally, some authors have shown that CR induces a redistribution of fat content away from the visceral towards the subcutaneous compartment^33^,^34^. Another hypothesis could be the browning of fat depots^35^, which could be responsible for changes in the endocrine and metabolic trends of fat tissue. However, we did not perform the experiments to validate these hypotheses in our model. Nonetheless, it may be argued that, besides a global change in adiposity, a reduction in visceral fat mass or browning of white adipose tissue may explain the beneficial effect of CR in our model.

Our first aim was to evaluate the effects of short-term moderate CR on SL mice programmed for increased cardio-metabolic risk by postnatal overfeeding. CR in NL groups was considered a necessary control for the supposedly universal beneficial effects of CR. The results we obtained were somewhat unexpected: CR did not deteriorate the entire metabolic phenotype of the NL mice, but the effects contrasted with those we observed in SL mice, for which CR improved all of the features observed. The finding that body fat percentages were increased, leptin levels were higher and cardiac oxidative stress augmented in NLCR mice was indeed surprising and not in agreement with other publications. For instance, Mitchell *et al*. observed the effects of graded levels of CR or protein restriction in mice^36^, and reported a reduction in circulating levels of leptin and insulin related to the grading of CR. In this study, oxidative stress was evaluated in the liver and in the plasma; however, the effect of CR on the heart was not studied. In their model, plasma antioxidant potential and reactive oxygen metabolites were not affected by CR and the changes in enzymatic antioxidant activity in the liver following CR were not significantly modified. There was also no indication that CR had an effect on oxidative damage in the liver. Indeed, in the literature “calorie restriction” in mammals refers to a wide range of protocols. The consequence of this diversity is that experimental results cannot be compared adequately. Since the amount of calories and levels of macro and micro-nutrients were restricted in our diet to a similar extent, “dietary restriction” or “food restriction” may be more appropriate term^37^. However, “calorie restriction” is the most common term used.

Concerning glucose metabolism, fasting blood glucose was not higher in adult SL mice; however, insulin levels were significantly increased, leading to a rise in HOMA-IR. Interestingly, our moderate CR reduced insulin levels and HOMA-IR in SL mice but not in NL mice. Glucose and insulin tolerance tests indicated that 6-month-old SL mice presented impaired glucose tolerance and insulin resistance. Hyperglycemia and hyperinsulinemia were reported in young SL mice at weaning^38^ with increased insulin expression in the pancreas. Hyperinsulinemia was also observed in adult SL rats^8^, with increased insulin secretion, accompanied by elevated GLUT2 content in pancreatic islets. These findings indicate that early postnatal overnutrition during a critical period of development may program permanent modifications in pancreas endocrine function and in glucose-stimulated insulin secretion. Moderate CR induced a beneficial effect on glucose metabolism in SL mice. After 4 weeks of CR, SL mice were able to regulate their glycaemia after the injection of glucose or insulin, while glucose intolerance and insulin resistance persisted in SLAL mice. CR did not modify glucose metabolism in NL mice. In postnatally overfed rats, CR from weaning^23^,^24^ reduced body-weight gain and normalized serum leptin and insulin patterns. However, only more severe and longer-term CR (3 months) was able to induce long-lasting effects on the regulation of body weight and of serum leptin and insulin, through a reversion of the early effects of programming on the hypothalamic appetite regulation system^24^.

Clinical studies have highlighted the major impact of early nutrition during childhood on long-term cardiovascular health^1^,^5^. In the present study, early postnatal over-nutrition led to impaired LVEF with enlarged left ventricular diameters in 6- and 7-month-old SL mice, which is consistent with our previous research. Indeed, we have shown that SL mice developed dilated cardiomyopathy at 6 months old, concomitant with an increase in collagen deposition and expression/activity of matrix-metalloproteinase-2 in the heart^10^. Indeed, alterations in the architectural organization in hearts and changes in ventricular compliance could explain the impairment of ventricular ejection fraction and the enlargement of ventricular diameter^14^. Cardiac remodeling can be triggered by hormones, cytokines, and growth factor-signaling pathways. In the heart, leptin or insulin may be considered either metabolic or growth factors for the heart and hyperinsulinemia and hyperleptinemia are documented in SL rodents at several stages of development.

Increasing evidence suggests that CR has pleiotropic effects on the cardiovascular system. Several studies have reported that CR significantly decreases oxidative damage in the aged heart^39^. We therefore aimed to determine whether CR could reverse cardiac alterations induced by postnatal overnutrition. Surprisingly, our research found that CR could ameliorate contractile function since LVEF was increased and left ventricular size decreased in SLCR mice, whereas CR did not improve systolic function in NL mice. Previous studies in humans have shown that CR could improve left ventricular diastolic function^40^ and that major risk factors for coronary heart disease are substantially improved in non-obese human beings^41^. In obese rats, CR attenuated not only obesity and hypertension, but also LV hypertrophy, fibrosis, and diastolic dysfunction^42^.

We therefore examined the recovery of pre-ischemic coronary flow, ventricular developed pressure, contractility and relaxation after I/R injury induced *in vitro* in isolated hearts, without any potential influence of neuro-humoral regulatory pathways. These results revealed that hearts from adult SL mice were more prone to ischemia-reperfusion injury with a markedly greater area of infarct, which is consistent with our previous studies^10^. A recent study showed that adult SL mice had significant alterations in heart metabolism proteins and a reduction in their efficiency after ischemia-reperfusion^43^. We also confirmed increased levels of oxidative stress in adult SL myocardial tissue, which is specifically reduced by CR in these mice. Our results showed that CR afforded protection against ischemic injury in SL as well as NL mice, with better recovery of LV developed pressure, max dP/dt, min dP/dt and diastolic pressure during post-ischemic reperfusion. These positive effects could be related to the induction of protective pathways with increased phosphorylation of STAT3. However, a markedly decreased area of infarct was observed only in SLCR mice, which is consistent with the improvement in LVEF *in vivo* and a lower cardiac oxidative stress level only in SLCR mice. Overall, these results emphasized the beneficial cardioprotective effect of CR in SL mice. A recent study showed that a 30% CR for 7 days was associated with cardio-protection, as evidenced by decreased infarct size in 4-month-old mice^44^. mRNA and microRNA analyses in heart tissue showed that genes modulated by short-term CR were associated with the circadian clock, oxidative stress, immune function, apoptosis, metabolism, angiogenesis, the cytoskeleton and extracellular matrix.

The heart exhibits highly aerobic metabolism due to the abundance of large mitochondria, which produce huge amounts of ATP necessary for continuous contraction. Mitochondria play an essential role in translating the early stress associated with fetal programming into cellular dysfunction observed in later life^45^. We therefore determined in adult SL mice whether mitochondrial function was modified by early overnutrition. Although a previous study showed mitochondrial ultrastructural abnormalities in mice fed a postnatal high-fat diet^25^, high-resolution respirometry showed no specific changes in cardiac muscle mitochondrial respiration induced by PNOF in adult SL mice, despite increased expression of proteins from complex IV and V. Therefore, it might be hypothesized that the efficiency of complexes IV and V is reduced in mice from SL groups. Conversely, CR seemed to exert a more significant effect on mitochondrial function: increase in states 2 (CI and CII), 3 and 4 (CV) of mitochondrial respiration in SLCR mice, while only state 3 was increased in NLCR mice. In parallel, only the increase of the expression of the complex I in SLCR specifically correlates with its increased respiration. Overall, these results highlight that, during CR, cardiac tissue increases the use of substrates to preserve its energy efficiency in SLCR mice, whereas only the coupling efficiency (state 3) is increased in NLCR mice, with an increased content of complex V. This fundamental difference might explain, at least in part, the differences of fat gain between the mice.

Mitochondria are also a major source of reactive oxygen species (ROS) as a by-product of the normal functioning of the respiratory chain. In cardiomyocytes, which have a high energy demand, a chronic state of oxidative stress exists^46^. Mitochondria, which play a central role in complex processes such as apoptosis, calcium homeostasis, ATP-production or oxidative stress, are centrally involved in many aspects of CR-induced protection against ischemic injury. Heart tissue from SL mice showed increased production of RONS, which was not counterbalanced by increased expression of antioxidant enzymes. After one month of CR, cardiac nitro-oxidative stress was attenuated in SL mice, while substrate-driven mitochondrial respiration was increased in all states, despite no specific increase in the expression of mitochondrial chain complexes (except for complex I). These results might be related to a greater efficiency of mitochondrial complexes, with a better transfer of electrons along the chain, associated with a decrease in cardiomyocyte RONS production. This could account for the CR-induced improvement in cardiac function and protection against ischemic injury.

In conclusion, our results confirmed the programming effect of early overnutrition on cardio-metabolic function. This study demonstrated that SL mice are not only moderately overweight but also present a cluster of metabolic modifications, such as fat accumulation, insulin resistance and glucose intolerance at 6 months old. In addition, SL mice presented major cardiac dysfunction with left ventricular dilation, lower LVEF, higher cardiac oxidative stress and hyper-sensitivity to ischemic-reperfusion injury. Short-term moderate CR not only normalized body weight in SL mice but also improved insulin- and glucose-sensitivity. Moreover, CR reversed the cardiac morphological and functional alterations induced by early overnutrition, attenuated cardiac RONS production, and improved mitochondrial oxidative efficiency. More studies are needed to confirm the exact pathways involved in this reduction in cardio-metabolic risk factors, but these results suggest that appropriate dietary behavior in adulthood could ameliorate metabolic programming and reverse the cardio-metabolic and oxidative dysfunction induced by early overnutrition.

## Materials and Methods

### Animals, groups and diets

All animals received humane care and study protocols complied with the institution’s guidelines. The investigation was carried out in accordance with Directive 2010/63/EU of the European Parliament and to the Guide for the Care and Use of Laboratory Animals published by the US National Institutes of Health (NIH Publication No. 85-23, revised 1996) and was approved by the local ethics committee (Comité d’Ethique de l’Expérimentation Animale Université-Bourgogne-Franche-Comté, Dijon, France, protocol agreement number: 00412.03), which specifically approved this study. Throughout the procedure, care was taken to avoid suffering and to ensure animal welfare, for instance, through improving the environment in cages. The safety and health of the animals were monitored every two days. During the protocol, no animals became moribund and needed to be killed early or died from the treatment. No specific signs of pain or distress (abnormal animal behavior, decreased food or water consumption, prostration) were observed throughout the study.

Adult female C57BL/6 mice (Charles River, L’Arbresle, France) were mated with male mice then housed in individual cages and given water and a standard pellet diet (A03, SAFE Diets Augy, France) *ad libitum* during pregnancy and lactation. To perform all the experiments presented in this study, a total of six consecutive series of reproduction were used; corresponding to 67 different litters. Female and male spawners participated in 3 different series of reproduction, and were then sacrificed. Therefore, 22 pregnant females were used in the study. On the third day of life, male pups were randomly distributed among mothers to achieve cross fostering and the litter size was adjusted to 9 male pups (normal litter, NL group), or reduced to 3 male pups in order to induce postnatal overfeeding (small litter, SL group). Each litter included pups from one to six different dams, which increased genetic variability within the litter. Excess pups were rapidly killed by decapitation after brief isoflurane anesthesia.

After weaning (day 24), mice of both groups had free access to a standard diet (A04, SAFE Diets Augy, France: 19.3% proteins, 8.4% lipids, 72.4% carbohydrates) and water. Throughout life, body weight and food intake were measured weekly then monthly. At 6 months of age, mice were randomly assigned to either the *ad libitum* (AL) or the CR groups. For the CR groups, the daily food supply was reduced by 20% (based on the food intake of each group) in normal litter (NLCR) and small litter group (SLCR), for one month, until the mice reached 7 months old. This short-term moderate CR did not induce micronutrient deprivation as severe 40% CR^47^. The remaining NL and SL mice continued to eat AL rodent chow (NLAL and SLAL).

### Body composition

Whole body composition (fat mass, lean mass and water) was determined at 7 months in awake mice by using nuclear magnetic resonance technology with an EchoMRI-700^TM^ instrument (EchoMedical Systems, Houston, TX, USA).

### Glucose (GTT) and Insulin (ITT) Tolerance Tests

Intraperitoneal GTTs and ITTs were performed in awake mice at 6 and 7 months of age, respectively (before and after 1-month of CR), after a 5-h fasting period. For GTTs, an intraperitoneal injection of glucose (2 g/kg) was administered. Blood droplets were collected from the tail vein just prior to the glucose injection (time 0) and at 15, 30, 45, 60, 75, 90, 105 and 120 min following the injection. The blood glucose level was measured using a glucometer and FreeStyle optium test strips (Abbott,USA). For ITTs, the mice were injected with 0.75 IU/kg human recombinant insulin (Actrapid^®^, Novo Nordisk, France) and blood glucose was determined at 0, 15, 30, 45, 60, 75, 90, 105 and 120 min.

### Echocardiography

As previously described^19^, transthoracic echocardiography was performed in 6 and 7-month-old mice before and after 1 month of CR or AL feeding, using the Vevo770™ imaging system (VisualSonics Inc., Toronto, Canada), equipped with a 30 MHz probe. Briefly, the mice were anesthetized with isoflurane, the body temperature of the mice was monitored using a rectal probe, and an infrared heating lamp was used to maintain body temperature at 37 ± 0.5 °C throughout the procedure. An electrocardiogram (ECG) signal was also monitored through electrode pads on the heated platform. Chest hair was removed using a chemical depilator to minimize ultrasound attenuation. The ultrasound probe (RMV-707B) was placed on the chest of the mice using warm ultrasound gel as a coupling medium.

Two-dimensional images were recorded in the parasternal long- and short-axis views to guide M-mode recordings obtained at the mid-ventricular level, with 3 to 5 measurements for each view in order to make an average. Wall thicknesses of the inter-ventricular septum (IVS) and left ventricular posterior wall (LVPW), as well as the LV diastolic and systolic internal dimensions (LVEDD and LVESD, respectively) were measured, and the average values were reported. LV systolic function was computed from the M-mode measurements according to the recommendations of the American Society of Echocardiography Committee. Left ventricular mass normalized to body weight (LVMN) was calculated using the formula:


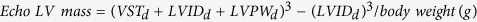


Pulsed Doppler studies of LV diastolic function were performed in the apical 4-chamber view with the Doppler cursor oriented parallel to the long-axis plane of the left ventricle. The sample volume was placed just below the level of the mitral valve and adjusted to render the highest early diastolic flow velocity peak of the transmitral Doppler flow signal. There was often a need for angle correction which was always less than 20 degrees. The early and late diastolic peak velocity (E, A) and their ratio (E/A) were derived from the transmitral Doppler waveform. Left ventricular systolic intervals of the isovolumic contract time (IVCT), the ventricular ejection time (ET) and diastolic intervals of the isovolumic relaxation time (IVRT) were also derived from the transmitral Doppler waveform. The myocardial performance index (Tei index) was calculated using the formula:


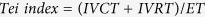


### Susceptibility to *ex vivo* Ischemia/Reperfusion (I/R) Injury

#### Preparation of isolated hearts

At 7 months of age, the mice were anesthetized with pentobarbital (80 mg/kg, i.p.) mixed with heparin (500 IU/kg, i.p) until total loss of nociceptive reflexes (verified by paw pinching). The hearts were excised and placed in a cold (4 °C) perfusion buffer bath until contractions ceased. The hearts were cannulated in the Langendorff mode and retrogradely perfused with Krebs-Henseleit buffer containing (in mmoles/L) 118 NaCl, 4.7 KCl, 25 NaHCO_3_, 1.2 KH_2_PO_4,_ 1.2 MgSO_4_·7H_2_O, 2.5 CaCl_2_·2H_2_O and 11 glucose equilibrated with 95% O_2_ and 5% CO_2_ at 37 °C [4].

Coronary flow, heart rate (HR) and left ventricular pressures: left ventricular end-diastolic pressure (LVEDP), left ventricular end-systolic pressure (LVESP), left ventricular developed pressure (LVDP = LVESP-LVEDP), and the first derivative of LVDP, left ventricular maximal pressure development (+dP/dt) and relaxation (-dP/dt) were recorded throughout the experiments (PowerLab®, LabChart® System, ADInstruments, Paris, France).

#### I/R protocol

The isolated hearts were initially perfused in the Langendorff mode for 15–20 min of stabilization. Cardiac parameters were then recorded. After 10 min, the perfusion was turned off in order to induce 30 min of total global normothermic ischemia, which was followed by reperfusion in the Langendorff mode for 2 hours.

#### Measurement of the infarct size

At the end of 2 h of reperfusion, the hearts were cut into five transverse slices, each about 1 mm thick. These slices were then incubated in triphenyl tetrazolium chloride (TTC) solution in phosphate buffer (pH 7.4, 37 °C) for 12 min and conserved in formalin. The slices were photographed from both sides and contoured with image analysis software (ImageJ) to delineate the borders of the entire heart and the infarcted area (white). The infarct size was calculated as a percentage of the total myocardial area.

### Plasma and Heart Sampling

At 7 months old, after 6 h fasting, mice were anesthetized by an intraperitoneal injection of sodium pentobarbital (80 mg/kg) mixed with heparin (500 IU/kg) until total loss of nociceptive reflexes (verified by paw pinching). Blood was collected from the heart, and immediately centrifuged at 4 °C in order to separate the plasma.

The heart was quickly harvested, the ventricles were separated from the atria and immediately frozen in liquid nitrogen. Tissue homogenates were prepared using a Precellys® 24 homogenizer (Bertin Technologies, Montigny-le-Bretonneux, France) under a controlled cooled temperature (Cryollys®, Bertin Technologies, Montigny-le-Bretonneux, France) in different appropriate reagents for RNA or protein extraction, and stored at −80 °C.

### Biochemical Measurements

Plasma glucose concentrations were determined using a glucometer, and plasma insulin and leptin were determined by EIA using specific commercial kits (Biorbyt, Cambridge, UK). Insulin resistance was estimated using the homeostasis model (HOMA) with the following formula: Insulin resistance (IR) = [fasting glucose (nM) × fasting insulin (mU/l)]/22.5^48^. Plasma adiponectin was determined by EIA using specific commercial kits (R&D Systems Bio-techne).

### Mitochondrial Respiration

#### Preparation of cardiac muscle fibers

Papillary muscle (2–3 mg of wet weight) was excised from fresh heart and put into cooled relaxing and preservation solution, BIOPS (in mM: CaK_2_EGTA 2.77, K_2_EGTA 7.23, MgCl_2_ 6.56, dithiothreitol 0.5, K-MES 50, imidazole 20, taurine 20, Na_2_ATP 5.77, phosphocreatine 15, pH 7.1 adjusted at 25 °C). The muscle fibers were separated from each other using sharpened forceps to leave only small areas of contact. The fibers were then transferred into vessels with cooled (in ice) BIOPS containing 50 μg/mL of saponin and incubated for 30 min with gentle stirring for complete solubilization of the sarcolemma. Permeabilized (skinned) fibers were then washed 3 times for 10 min in Mitochondrial Respiration Medium, MIR06 (in mM: EGTA 0.5, MgCl_2_ 3.0, K-lactobionic acid 60, taurine 20, K_2_HPO_4_ 10, HEPES 20, Sucrose 110 and BSA 1 g/L, Catalase 250 U/ml, pH 7.1 adjusted at 25 °C)^49^.

#### Respirometry protocol

The respiratory rates of cardiac fibers were determined with the Oroboros 2k-Oxygraph (Oroboros Instruments, Innsbruck, Austria) in 2 ml of MIR06 at 37 °C with continuous stirring. Before placing the tissue in the chamber, wet weight measurements were taken and a sample of 2–3 mg was used per chamber. Datlab software (Oroboros Instruments, Innsbruck, Austria) was used for data acquisition and analysis. Oxygen consumption rates were expressed as pmol of O_2_ per s per mg wet weight. Mitochondrial respiration was stimulated by the successive addition of substrates 10 mM glutamate, 2 mM malate, 5 mM pyruvate for complex I and 10 mM succinate for complex II to achieve the apparent state 2 (substrate-driven respiration). Then, 1.25 mM ADP was added to achieve the apparent state 3 respiration (maximal coupling respiration). Next, 0.75 mM carboxy-atractyloside (Catr) was added to block complex V and then ATP synthesis to achieve the apparent state 4 respiration (non-coupled respiration, also called maximal leak). Media were prepared and the protocol designed according to the guide provided by Oroboros Instruments. Technical sheets are available on the company Web site at http://www.oroboros.at/.

### Measurement of Reactive Nitrogen-oxygen Species in the Heart Using ESR Spectroscopy

Freshly harvested hearts were perfused with ice-cold Krebs-Henselheit buffer to remove residual blood. Myocardial biopsies of 3 mm diameter were taken from the apex of the left ventricle, rinsed, weighed and placed in a 24-well tissue culture plate containing 200 μL of ESR-specific pH 7.4 Krebs-HEPES buffer, to which deferroxamine (25 mM) and diethyl-dithiocarbamate (5 μM) were added to remove traces of contaminant metal ions. The spin probe 1-hydroxy-3-methoxycarbonyl-2,2,5,5- tetramethyl pyrrolidine (CMH) was added in order to reach a final concentration of 100 mM, then the tissue was incubated for 15 min at 37 °C, in a 5% CO_2_ atmosphere, in the presence of CMH^10^. In the presence of reactive nitrogen-oxygen species, the ESR-silent CMH hydroxylamine is oxidized into CP^•^ nitroxide radical. The buffer and tissue were collected and immediately frozen in liquid nitrogen then analyzed at 100K in a Bruker X-band EMX spectrometer (Wissembourg, France) using an HS cavity. The height of the central anisotropic peak of the CPN signal was measured (in arbitrary units, AU) and the results were expressed in AU/mg of fresh tissue.

### Real-time Quantitative PCR

Total RNA was extracted from frozen heart ventricular samples by Qiazol reagent (Invitrogen, Life Technologies, Saint Aubin, France). mRNA (1 μg) was reverse transcribed with MMLV reverse transcriptase (Invitrogen, id.) using random hexamers (Invitrogen, id.) according to the manufacturer’s instructions. Real-time quantitative polymerase chain reaction (RT-PCR) was performed with 2 μl of cDNA using the SYBR-Green PCR Master-Mix (Applied Biosystems) and both sense and antisense primers (5 mM) in a final volume of 20 μL, in a StepOne-Real-Time PCR system (Applied Biosystems). The primers used for the amplification of mouse genes are provided in Table 5. Target gene expression was normalized against the gene expression of housekeeping hypoxanthine-guanine phosphoribosyltransferase (HPRT).

### Western Blotting

Ventricular tissue was homogenized in 10 μl radio-immunoprecipitation assay (RIPA) buffer per mg wet weight and centrifuged at 5,000xg for 15 min at 4 °C. Proteins (40 μg) in the supernatant were separated using SDS-polyacrylamide gel electrophoresis under reducing and denaturing conditions and transferred to a 0.45 μm polyvinylidene difluoride membrane. The membrane was blocked with tris-buffered saline (TBS)-Tween, containing 5% (w/v) skimmed milk, before incubation overnight (4 °C) with a primary antibody diluted in TBS-Tween containing either 5% (w/v) skimmed milk or 5% (w/v) BSA. The primary antibodies used included a cocktail of antibodies that recognize the five respiration chain complexes, anti-OXPHOS (1:500, Abcam), anti-signal transducer and activator of transcription 3 (STAT3;1:2,000), anti-phospho-STAT3 for leptin signaling (pSTAT3; 1:2,000). For whole heart tissue, glyceraldehyde-3-phosphate dehydrogenase (GAPDH) was used as a loading control (1:1,000). All antibodies were obtained from Cell Signaling Technology (Beverly, MA, USA) unless otherwise specified. After incubation with the primary antibodies, blots were incubated with either anti-mouse (1:10,000) or anti-rabbit (1:2,000) IgG horseradish peroxidase-linked antibodies for 60 min at room temperature. This was followed by three 10-min washes in TBST and incubation in Clarity western ECL substrate chemiluminescent detection reagent (Bio-Rad) for 5 min prior to image acquisition. The chemiluminescent blots were first imaged with the ChemiDoc XRS + imager (Bio-Rad) and the band analysis tools of Image Lab software version 4.0.1 (Bio-Rad) were then used to select and determine the background-subtracted density of the bands. All protein was quantified to GAPDH and then the ratio of pSTAT3 to STAT3 was calculated.

### Statistical Analysis

Data are presented as mean ± standard error of mean (SEM). Categorical variables were compared by the chi-square test or Fisher’s exact test. For continuous variables, the normality of distribution was tested by the Shapiro-Wilk test. Student’s t test was used for 2-group comparisons of normally distributed data and the Mann-Whitney rank sum test for skewed data. Statistically significant differences between mean values for the four groups were tested by analysis of variance (two way ANOVA) followed by Tukey’s test. An ANOVA for repeated measurements followed by a Tukey post-hoc analysis was applied for data consisting of repeated observations at successive time points. The difference was considered significant when P < 0.05. The data were analyzed using SigmaPlot version 12.3.

## Additional Information

**How to cite this article**: Li, N. *et al*. Short-term moderate diet restriction in adulthood can reverse oxidative, cardiovascular and metabolic alterations induced by postnatal overfeeding in mice. *Sci. Rep.* **6**, 30817; doi: 10.1038/srep30817 (2016).

## Figures and Tables

**Table 1:** Daily food consumed, daily calories consumed and daily food consumed normalized to rodent body weight in NL and SL mice groups with or without calorie restriction (CR).

	NLAL (n = 30)	NLCR (n = 30)	SLAL (n = 29)	SLCR (n = 33)
Daily food consumed (g/mouse/day)	4.2 ± 0.5^a^	3.4 ± 0.2^b^	5.0 ± 0.8^c^	4.1 ± 0.2^a^
Daily calories consumed (Kcal/mouse/day )	13.4 ± 1.6^a^	10.9 ± 0.6^b^	16.1 ± 2.6^c^	13.1 ± 0.6^a^
Daily food consumed (g) normalized to body weight (g)	0.13 ± 0.01^a^	0.11 ± 0.01^b^	0.14 ± 0.01^a^	0.11 ± 0.01^b^

Significance was calculated by ANOVA test.

Means with distinct letters significantly differ, p < 0.05.

**Table 2:** Fasting blood parameters in NL and SL mice groups with or without calorie restriction (CR).

	NLAL (n = 9)	NLCR (n = 9)	SLAL (n = 10)	SLCR (n = 10)
Glucose (g/l)	1.98 ± 0.09^a^	1.65 ± 0.011^b^	1.99 ± 0.010^a^	1.62 ± 0.09^b^
Insulin (ng/ml)	0.98 ± 0.17^a^	0.95 ± 0.06^a^	1.59 ± 0.2^b^	0.59 ± 0.17^a^
HOMA-IR	11.40 ± 2.11^a^	8.58 ± 1.50^a^	17.18 ± 1.49^b^	5.47 ± 1.43^a^
Leptin (pg/ml)	62.8 ± 5.1^a^	131.3 ± 18.1^b^	151.8 ± 30.6^b^	140.9 ± 16.7^b^
Adiponectin (μg/ml)	1.48 ± 0.44^a^	1.59 ± 0.58^a^	1.57 ± 0.22^a^	1.77 ± 0.45^a^

Fasting blood glucose was assayed by glucometry; Plasma insulin, leptin and adiponectin and were estimated by ELISA. HOMA-IR = [fasting glucose (nM) × fasting insulin (mU/l)]/22.5.

Significance was calculated by ANOVA test.

Means with distinct letters significantly differ, p < 0.05.

**Table 3:** Echocardiographic measurements in mice at 6 and 7 months old.

n	6 months old (before CR)				7 months old (after CR)			
	NLAL	NLCR	SLAL	SLCR	NLAL	NLCR	SLAL	SLCR
	20	10	19	21	20	10	19	21
HR, bpm	438 ± 52	363 ± 36*	409 ± 39	423 ± 52	449 ± 49	375 ± 15*	459 ± 40^†^	413 ± 58*
LVEF, %	57 ± 6	58 ± 3	50 ± 6^ǂ^	52 ± 4^ǂ^	59 ± 4	60 ± 4	49 ± 5^ǂ^	60 ± 6*^†^
LVEDD, mm	4.22 ± 0.26	3.95 ± 0.14	4.45 ± 0.18^ǂ^	4.48 ± 0.26^ǂ^	4.22 ± 0.26	4.06 ± 0.09	4.53 ± 0.1^ǂ^	4.17 ± 0.31*^†^
LVMN, mg/g	3.15 ± 0.81	3.71 ± 0.35	3.63 ± 0.51	3.96 ± 0.56^ǂ^	3.81 ± 0.54^†^	3.68 ± 0.35	3.69 ± 0.55	4.12 ± 0.9
E/A ratio	1.36 ± 0.21	1.56 ± 0.22	1.58 ± 0.24	1.76 ± 1.26	1.34 ± 0.12	1.49 ± 0.16	1.50 ± 0.50	1.73 ± 0.48
E/E'	39 ± 5	40 ± 9	39 ± 10	37 ± 9	41 ± 8	44 ± 11	43 ± 11	47 ± 8
Tei index	0.63 ± 0.13	0.70 ± 0.11	0.64 ± 0.10	0.75 ± 0.18	0.76 ± 0.08	0.75 ± 0.15	0.71 ± 0.10	0.71 ± 0.10

Data are means ± standard deviation. Bpm indicates beats per minute; HR: heart rate; LVEF: left ventricular ejection fraction; LVEDD: left ventricular end-diastolic diameter; LVMN: left ventricular mass normalized to body weight; n: number of mice.

*p < 0.05 significant difference in comparison with corresponding AL group.

^†^p < 0.05 significant difference in comparison with the same group at 6 months old.

^ǂ^p < 0.05 significant difference in comparison with NLAL group at the same age.

**Table 4:** Antioxidant enzyme gene expression (by DDCt) in hearts of NL and SL mice with or without calorie restriction (CR).

Gene	NLAL (n = 10)	NLCR (n = 10)	SLAL (n = 10)	SLCR (n = 10)
MnSOD	0.99 ± 0.09^a^	0.91 ± 0.04^a^	1.09 ± 0.04^a^	0.98 ± 0.03^a^
Cu/ZnSOD	1.14 ± 0.09^a^	1.15 ± 0.14^a^	1.11 ± 0.10^a^	1.12 ± 0.11^a^
Catalase	0.80 ± 0.08^a^	0.96 ± 0.16^a^	0.99 ± 0.11^a^	0.99 ± 0.13^a^

Significance was calculated by ANOVA test.

(MnSOD: manganese-superoxide dismutase, Cu/ZnSOD: copper/zinc superoxide dismutase).

Means with distinct letters significantly differ, p < 0.05.

**Table 5:** Forward and reverse sequences of primers used for the amplification of mouse genes.

Gene	Forward (5′ to 3′)	Reverse (5′ to 3′)
HPRT	CTGGTGAAAAGGACCTCTCG	TGAAGTACTCATTATAGTCAAGGGCA
MnSOD	AACTCAGGTCGCTCTTCAGC	GCTTGATAGCCTCCAGCAAC
Cu/ZnSOD	CTGCTCGCTCACATAACAGC	ATGGCTGAGGTTCTCTGCAC
Catalase	GATGAAGCAGTGGAAGGAGC	ACCACATCCTGAACGAGGAG

(HPRT: hypoxanthine-guanine phosphoribosyltransferase, MnSOD: manganese-superoxide dismutase, Cu/ZnSOD: copper/zinc superoxide dismutase).
